# Effects of an Inquiry-Based Short Intervention on State Test Anxiety in Comparison to Alternative Coping Strategies

**DOI:** 10.3389/fpsyg.2018.00201

**Published:** 2018-02-20

**Authors:** Ann Krispenz, Oliver Dickhäuser

**Affiliations:** School of Social Sciences, Department of Psychology, University of Mannheim, Mannheim, Germany

**Keywords:** educational psychology, test anxiety, cognitive appraisals, inquiry-based stress reduction, short intervention

## Abstract

**Background and Objectives:** Test anxiety can have undesirable consequences for learning and academic achievement. The control-value theory of achievement emotions assumes that test anxiety is experienced if a student appraises an achievement situation as important (value appraisal), but feels that the situation and its outcome are not fully under his or her control (control appraisal). Accordingly, modification of cognitive appraisals is assumed to reduce test anxiety. One method aiming at the modification of appraisals is inquiry-based stress reduction. In the present study (*N* = 162), we assessed the effects of an inquiry-based short intervention on test anxiety.

**Design:** Short-term longitudinal, randomized control trial.

**Methods:** Focusing on an individual worry thought, 53 university students received an inquiry-based short intervention. Control participants reflected on their worry thought (*n* = 55) or were distracted (*n* = 52). Thought related test anxiety was assessed before, immediately after, and 2 days after the experimental treatment.

**Results:** After the intervention as well as 2 days later, individuals who had received the inquiry-based intervention demonstrated significantly lower test anxiety than participants from the pooled control groups. Further analyses showed that the inquiry-based short intervention was more effective than reflecting on a worry thought but had no advantage over distraction.

**Conclusions:** Our findings provide first experimental evidence for the effectiveness of an inquiry-based short intervention in reducing students’ test anxiety.

## Introduction

Test anxiety is the emotion most prevalent in the educational context. For example, 15–20% of college students are assumed to suffer from test anxiety (Ergene, 2003). These are staggering facts, considering that test anxiety is negatively associated with academic achievement (e.g., Cassady and Johnson, 2002). Test anxiety has a negative impact on academic achievement for several reasons. First, test anxiety reduces the capacity of working memory by utilizing the resources of the central executive (e.g., Eysenck, 2010). As a result, learning within the context of complex or difficult tasks is impaired (Hodapp and Benson, 1997). Second, test anxiety decreases intrinsic learning motivation (Pekrun and Hofmann, 1999) and, as a consequence, impedes academic achievement for some students (Pekrun et al., 2002). Third, test anxiety can lead to the use of more surface level learning strategies (e.g., simple rehearsal of the study material), while it decreases the use of deep level learning strategies (e.g., elaboration of the study material). Helping individuals to deal with their test anxiety is hence an effort worth taking. The aim of the present paper is to investigate the effectiveness of a short inquiry-based stress intervention (IBSR; Mitchell and Mitchell, 2003) that allows self-exploration of test anxiety causing worry thoughts.

### Test Anxiety

Test anxiety is a prospective achievement emotion (Pekrun, 2006) regarding a future achievement outcome (e.g., passing an exam) as a result of an achievement activity (e.g., preparing for the exam). It can be defined as worry and agitation in an achievement context (e.g., exams or test) that is perceived as threatening for one’s own self-worth (Schwarzer, 2000). *Physiologically*, test anxiety is characterized by an increased state of arousal going along with sweating, palpitations, trembling, and nausea. Test anxiety is accompanied by an unpleasant *affective* state of agitation, feelings of insecurity, and helplessness. On a *cognitive* level, test anxiety is marked by specific thoughts (e.g., regarding the consequences of potential failure). *Motivationally*, test anxiety comes with certain tendencies to act (e.g., avoidance of an exam). Test anxiety often expresses itself through typical postures or mimics (e.g., worrisome facial expressions). In test anxiety research, the physiological and affective components are typically referred to as the emotionality component of test anxiety while the cognitive and the motivational components are labeled as the worry component.

### Control-Value Theory of Achievement Emotions

According to control-value theory of achievement emotions (Pekrun, 2006), test anxiety is mainly caused by cognitive mechanisms, in particular a combination of cognitive value and control appraisals. Cognitive appraisals are based on continuous, complex, and evaluative processes. They allow individuals to make distinctions between benign and dangerous situations (Lazarus and Folkman, 1984). *Value appraisals* concern the subjective value of achievement activities and their outcomes for the individual. *Control appraisals* refer to the subjective control (i.e., the individual perceives a specific degree of causal influence; Skinner, 1996) regarding these achievement activities and achievement outcomes. Accordingly, test anxiety is experienced if one evaluates the outcome of a testing situation (e.g., passing an exam) as subjectively highly important, but one is unsure if the achievement activities (e.g., preparing for the exam) will be successful to obtain the desired outcome (Pekrun et al., 2002). For example, a student will feel anxious if passing an exam is really important to her/him because otherwise she/he will not be able to get the job she/he wants (value appraisal), but if she/he is unsure if she/he will find enough time to prepare for the exam (control appraisal).

Appraisals are influenced, amongst other determinants, by the individual’s beliefs. How one appraises a specific testing situation depends on what one in general beliefs regarding similar testing situations (Pekrun, 2006). Value appraisals are shaped by beliefs concerning the relevance of achievement and its consequences (Pekrun, 1988). Beliefs leading to control appraisals include self-concepts, expectancies that an action (e.g., preparing for an exam) can be initiated and performed, expectancies that one’s actions will cause a certain (positive) action-outcome (e.g., passing the exam), and causal attributions (i.e., retrospective appraisals of the causes of academic success or failure). Hence, it is not only the objective circumstances of the testing situation, but also the subjective cognitive interpretation of these circumstances that lead to test anxiety.

### Cognitive Approaches to the Treatment of Test Anxiety

Control-value theory of achievement emotions (Pekrun, 2006) suggests – amongst others – a cognitive (i.e., appraisal-oriented) treatment of test anxiety. Accordingly, existing therapies strongly focus on the change of (irrational) beliefs and thought patterns to reduce test anxiety. For example, in rational emotive behavior therapy (Ellis, 2002), individuals are encouraged to question their own thinking patterns with techniques such as direct cognitive debate and logical persuasion in order to *replace* dysfunctional and irrational beliefs with more realistic ones. In systematic rational restructuring (Goldfried et al., 1974) – a further form of cognitive therapy – anxious participants are encouraged to *counter* their negative thinking with positive thoughts (e.g., the belief “I will fail” is transformed to “It is not terrible to fail, only inconvenient”).

Another potentially effective approach to modify cognitive appraisals is inquiry-based stress reduction (IBSR; Mitchell and Mitchell, 2003). In the first step of IBSR, a standardized procedure is applied to identify stressful cognitions causing anxiety (van Rhijn et al., 2015). In the second step of IBSR, each belief that has been identified is explored by means of four questions and several sub-questions. In this approach, the participant does not only reflect on the effects, causes, benefits, and functionality of the respective stressful cognitions, but is also enabled to perceive and experience reality without the distortions caused by the stressful cognitions. In the third and last step of IBSR, the participant is asked to explore if the opposite of the initial belief could also be valid. This should allow the participant to overcome his or her tendency to seek or interpret evidence in ways that are partial to existing beliefs (i.e., the confirmation bias; Nickerson, 1998) by finding evidence in support of the turnaround thought. Going through the IBSR process thus allows the participant to reevaluate his or her initial appraisals and, as a consequence, to change negative feelings (e.g., anxiety) following from the initial negative interpretation of the situation. As this reevaluation process is high in personal relevance, comprehensive and systematic, according to dual process models of information processing (e.g., the heuristic systematic model; Chen and Chaiken, 1999) the reevaluation and the associated emotional change should be lasting. This should especially be true since the new arguments leading to the reevaluation of the initial anxiety causing cognitions are self-created and, thus, even more convincing and belief changing (Briñol et al., 2012).

Inquiry-based stress reduction differs from other cognitive test anxiety interventions such as rational emotive behavior therapy (Ellis, 2002) and systematic rational restructuring (Goldfried et al., 1974) in several ways: First, and most importantly, IBSR does not employ a cognitive debate of participants’ dysfunctional and irrational beliefs through logical persuasion by a therapist, but supports *experiential* self-exploration. This way, participants’ irrational thinking is not replaced with more rational beliefs *per se* (as in rational emotive behavior therapy) or countered with positive thoughts (as in systematic rational restructuring). Rather, the IBSR method allows the participant to reinterpret the frightening situation according to his or her own inner wisdom (i.e., knowledge not restricted to logical analysis only but rather an integration of all kinds of knowing based on observation, kinesthetic and sensory experiences, behavioral learning and intuition; Linehan, 1993; van Rhijn et al., 2015). In addition to this core difference, there are also distinctions, which are important from a practical perspective: Due to the standardized IBSR procedure, the process of modifying irrational and negative thinking is guided by a simple and clear defined set of questions, allowing for a structured way of *self*-inquiry. As a consequence, the practice of IBSR does not require a therapeutic setting. This makes the application of the IBSR method not only available to individuals suffering from a psychological disorder (e.g., depression), but for everyone who wants to change their negative thinking in order to reduce anxiety (e.g., test anxious students).

Until now, research on IBSR as an intervention for test anxiety does not exist. However, there is first empirical evidence of the IBSR methods’ potential to reduce anxiety. For example, in a single-group study (*N* = 47), participants of a non-clinical sample received a 9-day IBSR intervention (Leufke et al., 2013). On the 1st day of the intervention, participants learned how to identify stressful cognitions and to investigate them using IBSR. During the following 8 days, participants focused on different stressful life events (e.g., death of a relative, breakup of a close relationships, loss of job, etc.) and used IBSR to identify and investigate their stressful thoughts related to these events. Participants’ anxiety (amongst other psychopathological symptoms) was measured before and immediately after the intervention. Also, there was a follow-up measure 3 months after the intervention. Results revealed that participants’ anxiety declined long-term (i.e., for at least 3 months). Using a similar single-group design, Smernoff et al. (2015) also found anxiety to decline in participants (*N* = 197) of a 9-day IBSR intervention and effects to remain stable at a 6-months follow-up evaluation. However, in both of these studies a control group was missing. This makes the interpretation of the results difficult, as effects might be due to other factors than the IBSR intervention. The results could, for example, be a simple effect of repeated measurement. The first measurement could have led to increased attention to the variables under consideration and this increased attention – but not the intervention – could have resulted in lower values on these variables at a later measurement. This limitation was overcome by a recent study (Krispenz and Dickhäuser, 2016). Using a non-clinical sample and applying a two-group design (intervention vs. matched control group), the authors were able to demonstrate that IBSR is potent in reducing trait anxiety (and chronic stress) long-term. In this study (*N* = 79), trait anxiety was assessed before and 3 months after a 9-day IBSR intervention. Results revealed that trait anxiety decreased over time for participants of the IBSR intervention group, but not for participants of the control group who had not received any intervention. However, the study design of Krispenz and Dickhäuser (2016) did not include an *active* control group allowing only for a weak comparison. Additionally, in none of the studies mentioned above, randomized assignment of participants to the intervention group was possible due to the self-enrolment process of IBSR participants, which might have resulted in a selection bias. Thus, results of these former studies can only be generalized for individuals interested and engaged in self-enhancement through interventions like IBSR. Finally, in all these former studies, participants received a 9-day IBSR intervention. However, the length of the intervention makes participation very time consuming, a possible obstacle preventing individuals from attendance.

### The Present Research

The present study significantly adds to the research on IBSR by overcoming these shortcomings. In a randomized control trial with a short-term longitudinal design, we investigated the effects of a 20 min IBSR short intervention on test anxiety in a sample of university students. This approach has the following advantages: For the first time, we investigated the effects of IBSR on test anxiety in an educational setting. Further, we randomly assigned the participants to the experimental conditions making a causal interpretation of effects possible. Also, we used two active control groups to allow for a strong comparison between the control groups and the intervention group. Finally, prior to study participation individuals were not aware that they would receive an IBSR intervention, preventing a potential self-selection bias.

The specific goal of our study was to investigate the effect of an IBSR short intervention on students’ test anxiety in relation to one individual worry thought. Participants of the IBSR group received a 20 min IBSR intervention to explore an individual worry thought. Participants of the control groups received no intervention but tasks mirroring strategies often used by anxious students. Participants of the first control group received a distraction task to mirror *mental disengagement*. Mental disengagement includes a wide range of activities (e.g., sleeping, watching TV, daydreaming), which help anxious individuals to take their minds off frightening thoughts (Carver et al., 1989). Through mental disengagement, anxiety can be reduced for a short period of time (Rost and Schermer, 2007). Participants of the second control group were asked to reflect on their worry thought to mirror the tendency to focus on worry thoughts and to *vent* the arising anxiety (Carver et al., 1989). Focusing and venting makes the worry thoughts more salient and should not reduce thought related test anxiety.

We had the following prediction: Participants receiving the IBSR intervention were assumed to experience less thought related test anxiety than participants of both control groups (*combined* analysis) immediately after and 2 days after the experiment. This prediction was based on the rationale that only IBSR – but neither mental disengagement nor venting – aims at the modification of cognitive appraisals (i.e., worry thoughts). Also, we expected distraction from the worry thought to have at least a short-term effect on thought related test anxiety (Rost and Schermer, 2007). However, since distraction works primarily through the use of attentional deployment (Thiruchselvam et al., 2011), but does not alter the interpretation of an emotional stimulus (i.e., the worry thought) we did not know how pronounced the anxiety reducing effect of distraction would be in comparison to the IBSR intervention. Thus, we additionally wanted to explore the effects of IBSR vs. distraction (i.e., mental disengagement) vs. reflection (i.e., venting) on thought related test anxiety (*differential* analysis).

## Materials and Methods

### Participants

A total sample of 162 students of the University of Mannheim, (*M*_age_ = 21.63, *SD* = 4.07, range = 18–55 years, 67.9% women) with different study subjects participated in the study in partial fulfillment of departmental requirements. 91.4% of the study participants reported German to be their first language. At the time, most participants studied psychology (48.1%) or other social sciences (24.1%) with a study duration of *M* = 3.69 semesters (*SD* = 1.72). Participants indicated to have at least two academic exams at the end of the same semester (*M* = 4.44 tests, *SD* = 0.91).

### Procedure

By the time we conducted the study and acquired the data, it was neither compulsory nor customary at the respective university to seek explicit ethical approval for an experimental study including only participants’ self-reports on test anxiety. However, we carefully ensured that the study was conducted in line with the ethical guidelines of the American Psychological Association and in full accordance with the ethical guidelines of the German Psychological Society. The study exclusively made use of anonymous questionnaires. The data was matched for the analyses using codenames only. Written informed consent was obtained according to the guidelines of the German Psychological Society. Informed consent included information about (1) research object, (2) study procedure, (3) duration and allowance, (4) possible benefits of participation, (5) anonymity of data collection, and (6) possible risks of participation. Also, participants were explicitly informed that participation was voluntary and could be terminated at any time without any reason or negative consequences for the participant. Participants had to declare that they were at least 18 years old, had read the informed consent information, and agreed to the rules of participation.

The study had two parts. The first part of the study took place in a university laboratory in individual sessions and lasted about 45 min. All measures and instructions were provided on a laptop. After giving written informed consent, participants’ trait test anxiety was measured as a control variable. Then, participants were asked to think of the upcoming academic exams and to consider which of these exams frightened them the most. Next, participants were asked to describe their most frightening exam in detail, to rate the exams’ personal value and their anxiety-level regarding this exam. Then, participants were asked to reflect what worried them the most regarding the upcoming and most frightening exam and to write it down in one sentence (individual worry thought). Finally, participants rated the test anxiety caused by that individual worry thought (thought related test anxiety). In the next step, participants were randomly assigned to the three experimental conditions.

Experimental conditions differed in the way, participants were asked to deal with their individual worry thought. Participants of the IBSR intervention group were explicitly instructed to self-explore their individual worry thought in a written form and by the use of the four questions and several sub-questions provided by the IBSR technique (see **Table 1**). As a first sub-step, the validity of the worry thought was questioned (Questions 1 and 2). Guided by Question 3 (“How do you react, what happens when you have this thought?”), participants then reported the mental pictures they associated with the worry thought and reflected on its emotional and behavioral effects. Further, participants explored and experienced the bodily sensations going along with the respective cognitions. After that and guided by Question 4 (“Who would you be without the thought?”), participants were enabled to perceive and experience reality without the distortions caused by the worry thought. Afterward, participants were instructed to turn their individual worry thought (e.g., “There is too much stuff to learn.”) around to the opposite (e.g., “There is *not* too much stuff to learn.”) and to find specific examples of how the turnaround could be valid for the situation they had described. Participants of the distraction control group were distracted from the worry thought by a transcription task. They received a text of 190 words about the university taken from Wikipedia and were instructed to copy the text on paper. Participants of the reflection control group were asked to report their reactions to their individual worry thought in writing to induce venting. Immediately after the experimental treatment, all participants were again asked to rate their thought related test anxiety. Also, participants of the IBSR intervention group were asked to report any problems they might have had with the application of the IBSR technique. Last, participants provided demographic data and generated a personal code for data matching.

**Table 1:** Inquiry-based stress reduction (IBSR) questions used in the present study.

Question	Format of answer
**Q1: Is this thought true?**	Yes vs. no
**Q2: Can you absolutely know that this thought is true?**	Yes vs. no
**Q3: How do you react, what happens when you have this thought?**	Open
Does that thought bring peace or stress to your life?	Open
What emotions arise when you have that thought?	Open
What physical sensations arise having these emotions?	Open
What images do you see, past or future, as you think this thought?	Open
How do you treat yourself when you have this thought?	Open
How do you treat others when you have this thought?	Open
Do any obsessions or addictions begin to appear when you have this thought (e.g., alcohol, drugs, shopping, food, television)?	Open
**Q4: Who would you be without the thought?**	Open

The second part of the study was conducted online approximately 2 days after the experimental part and lasted about 5 min. First, participants were instructed to remember their most frightening exam and their individual worry thought. Then, thought related test anxiety was assessed. After that, participants were debriefed.

### Adherence to Experimental Protocol

To ensure that participants were able to follow the experimental protocol, we provided clear instructions for each experimental condition. Also, there was always a member of the research team in the lab in case participants had questions regarding the experimental instructions. Additionally, two independent raters (the first author and one additional researcher familiar with IBSR) rated participants’ answers to the IBSR questions, thought protocols, and copy tasks. Possible ratings were binary with 1 (*adherence to experimental protocol*) and 0 (*deviation from experimental protocol*). For IBSR participants, deviation was coded when participants did not answer the IBSR questions in a meaningful way. For participants of the control groups, deviation was coded for participants who did not write a thought protocol or copy the respective text. The independent raters agreed 100% (Cohen’s kappa = 1.00, *p* < 0.001). Two cases of deviation from the experimental protocol were detected for the IBSR group: These two participants did not answer the IBSR questions in a meaningful way, but made remarks about the study design and reported to have experienced no test anxiety. As a consequence, both participants were excluded from the data analyses.

### Measures

#### Measures Related to the Most Frightening Exam

The exams’ personal value was assessed with one item (“The exam described above is…”). Ratings were made using a 10 points scale ranging from 1 (*not at all important to me*) to 10 (*extremely important to me*). Anxiety regarding the exam was assessed with one item (“The exam described above frightens me…”). Ratings were made using a 10 points scale ranging from 1 (*almost not at all*) to 10 (*extremely*).

#### Thought Related Test Anxiety

Thought related test anxiety was assessed with a slightly modified version of the German short version of the state scale of the State-Trait Anxiety Inventory (STAI-SKD; Bertrams and Englert, 2013). The STAI-SKD allows for the assessment of state test anxiety with three items covering the emotionality dimension (“I am tense,” “I am agitated,” “I am nervous.”) and two items more strongly corresponding to the worry dimension (“I am disturbed,” “I am worried.”). For the present study, participants were instructed to indicate how they felt when having an individual worry thought in regard to the STAI-SKD items (e.g., “Having this thought, I am tense”). Ratings were made using 4-point scales from 1 (not at all) to 4 (very much). Following the conception of this measure and in accordance with Bertrams and Englert (2013), we used a combined STAI-SKD mean score including all five items, with high scores indicating a high level of thought related test anxiety. The STAI-SKD items showed excellent internal consistencies (Cronbach’s α time 1 = 0.87, time 2 = 0.92, and time 3 = 0.92).

#### Trait Test Anxiety

As a control variable, trait test anxiety was measured using the short version of the German Test Anxiety Inventory (TAI-G; Wacker et al., 2008). The TAI-G allows for the standardized assessment of trait test anxiety with 15 items (e.g., “I am thinking about what happens if I fail”). Participants were instructed to indicate how they generally felt and what they were generally thinking in test situations in regard to the TAI-G items using a 4-point scale ranging from 1 (almost never) to 4 (almost always). After recoding reversed items, a mean trait test anxiety score was calculated, with high scores indicating high levels of trait test anxiety. The TAI-G items showed excellent internal consistency (Cronbach’s α = 0.89).

### Attrition and Missing Data

One hundred and sixty-two participants completed the baseline measures (*n* IBSR = 55 vs. *n* distraction = 52 vs. *n* reflection = 55) and the post measures in the laboratory without any attrition. One hundred and thirty-four participants answered follow-up measures (*n* IBSR = 46 vs. *n* distraction = 42 vs. *n* reflection = 46), which equals an attrition of 17.28% between times 2 and 3. Allover, missing data ranged from a low of 0.6% (participants’ age) to a high of 17.28% (thought related test anxiety). The frequency of missing data for all variables is reported in **Table 2**. To analyze the pattern of missing data, we used the approach suggested by Schlomer et al. (2010). First, we created a dummy variable (code 1 = missing, 0 = non-missing). Then, we empirically evaluated the relations between the observed variables and missing values. A multivariate analysis of variance with trait test anxiety, exams’ personal value, anxiety regarding the exam, and initial thought related test anxiety as dependent variables revealed no statistically significant overall multivariate effect of the dummy variable, *F*(4,157) = 0.70, *p* = 0.594, $\eta_{p}^{2}$ = 0.017. A statistically non-significant χ^2^-test also showed, that attrition rates did not systematically differ between groups (missing vs. non-missing), χ^2^(2) = 0.02, *p* = 0.991. These results indicate that missing data was missing completely at random (MCAR).

**Table 2:** Pattern of missing data.

	*N*	*M*	*SD*	Missing (*n*)	Missing (%)
Age	161	21.63	4.07	1	0.6
Sex	162			0	0
Language	162			0	0
Study subject	162			0	0
Study duration (Semester)	159	3.69	1.72	3	1.9
Number of exams	162	4.44	0.91	0	0

Trait test anxiety	162	2.35	0.57	0	0
Anxiety regarding exam	162	6.92	2.15	0	0
Exams’ personal value	162	7.88	1.83	0	0

**Thought related test anxiety**					
Pre-intervention	162	2.79	0.74	0	0
Post-intervention	162	2.49	0.84	0	0
Follow-up	134	2.31	0.78	28	17.3

In the following analyses, missing data was handled with the Full Information Maximum Likelihood Imputation provided by Mplus (Muthén and Muthén, 1998–2012). This approach was chosen because listwise deletion of missing data generally creates biased parameter estimates as well as biased significance testing (Schlomer et al., 2010).

### Data Analyses

#### Design

The study had a 3 × 3 mixed-factors design. Participants were randomly assigned to the three experimental conditions (IBSR intervention vs. distraction vs. reflection). Measures were taken pre-intervention (time 1), post-intervention (time 2) and approximately 2 days after the intervention (follow-up, time 3).

#### Path Analyses

We used path analyses to test our hypotheses. All analyses were conducted with the software Mplus (Muthén and Muthén, 1998–2012). We applied the robust MLR-estimator. Even though evaluation of model fit was not the focus of the present investigation and sample size was rather small, for the sake of complete reporting we also included fit indices following the guidelines given by Schermelleh-Engel et al. (2003).

##### Model 1: Combined analysis

**FIGURE 1:**
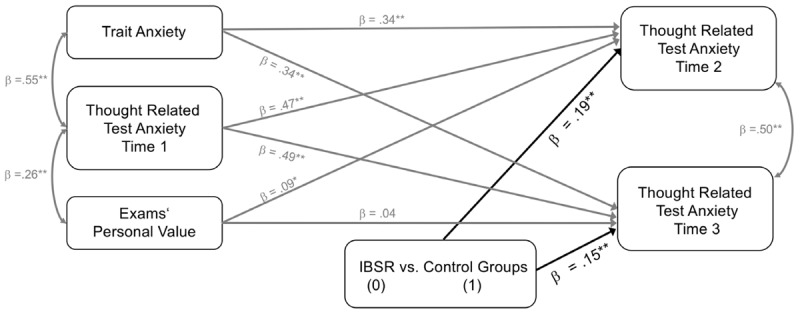
Experimental intervention effects on targeted thought related test anxiety at times 2 and 3 while controlling for initial scores of thought related test anxiety, trait test anxiety, and exams’ personal value. IBSR (dummy coded 0) vs. distraction and reflection group (both dummy coded 1). All parameter estimates are standardized. *N* = 160. ^∗^*p* ≤ 0.05, ^∗∗^*p* ≤ 0.01.

First, we investigated the effects of the IBSR intervention on thought related test anxiety measured at times 2 and 3 in comparison to both control groups. We expected individuals receiving the IBSR intervention to experience less thought related test anxiety than participants of the control groups for times 2 and 3. To test this hypothesis, we coded a dummy variable (d1) for which IBSR was selected as the reference group (coded 0), while the distraction and the reflection group (both coded 1) were contrasted with this reference group. In model 1 (**Figure 1**), thought related test anxiety measured at time 2 as well as at time 3 were both included as dependent variables with their residuals correlated to account for the non-independence of the dependent variables by avoiding an overly stringent autoregressive path from time 2 to time 3. Further, we included an autoregressive path from initial thought related test anxiety (measured at time 1) on both dependent variables, respectively. Also, we allowed for direct paths from the dummy variable d1 on both dependent variables, respectively. In addition, we assumed participants’ trait test anxiety to have a major positive influence on their thought related test anxiety at times 2 and 3, which is in accordance with the state-trait-model of anxiety (Spielberger and Vagg, 1995). Finally and in line with the assumptions of control-value theory (Pekrun, 2006), though related test anxiety at times 2 and 3 was expected to depend on exams’ personal value. For the same theoretical reasons, we also assumed correlations between the covariates (trait test anxiety, exams’ personal value) and thought related test anxiety at time 1.

##### Model 2: Differential analysis

**FIGURE 2:**
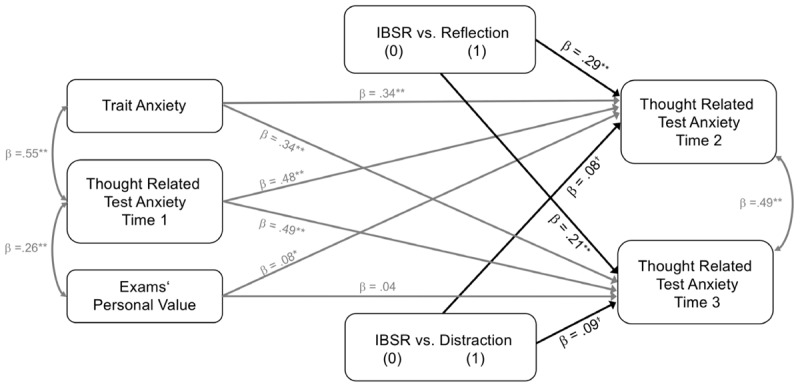
Experimental intervention effects on targeted thought related test anxiety at times 2 and 3 while controlling for pre-intervention scores of thought related test anxiety, trait test anxiety, and exams’ personal value. IBSR vs. the reflection (IBSR dummy coded = 0, reflection dummy coded = 1, distraction dummy coded 0); IBSR vs. the distraction (IBSR dummy coded = 0, reflection dummy coded = 0, distraction dummy coded 1). All parameter estimates are standardized. *N* = 160. ^†^*p* ≤ 0.10, ^∗^*p* ≤ 0.05, ^∗∗^*p* ≤ 0.01.

Second, we wanted to explore the effects of the IBSR intervention on thought related test anxiety at times 2 and 3 in comparison to both control groups, respectively. In particular, we wanted to explore whether IBSR is more effective in reducing thought related test anxiety than distraction from or than reflection on the worry thought. We hence coded two dummy variables (d2a and d2b). Again, we selected IBSR as the reference group (coded 0 in both dummy variables) and contrasted it with the respective control group (coded as 1). Thus, dummy variable d2a allows to compare the IBSR group with the reflection group (0 = IBSR, 1 = reflection, 0 = distraction) while dummy variable d2b compares IBSR with distraction (0 = IBSR, 0 = reflection, 1 = distraction). Except for the added dummy variable, model 2 (**Figure 2**) matches model 1 (**Figure 1**). We allowed for direct paths from both dummy variables on both dependent variables (thought related test anxiety measured at times 2 and 3), respectively.

## Results

### Descriptive Statistics

Participants (*n* = 160) reported a trait test anxiety level of *M* = 2.37 (*SD* = 0.56, range = 1.13–3.73) and an anxiety level regarding their most frightening exam of *M* = 7.00 (*SD* = 2.05, range = 1.00–10.00). Also, participants highly valued their most frightening exam (*M* = 7.93, *SD* = 1.75, range = 3.00–10.00) and reported an initial thought related test anxiety of *M* = 2.81 (*SD* = 0.71, range = 1.20–4.00).^1^ A multivariate analysis of variance with the factor Condition (IBSR vs. distraction vs. reflection) as independent variable and trait test anxiety, anxiety level regarding their most frightening exam, exams’ personal value, and initial thought related test anxiety as dependent variables revealed a non-significant overall multivariate effect, *F*(8,310) = 0.51, *p* = 0.850, $\eta_{p}^{2}$ = 0.01. Further, there were only non-significant univariate effects (all *p*s > 0.187), indicating that experimental conditions did not differ regarding baseline levels of the analyzed variables. Hence, randomization had been successful. Zero order correlations for the variables used in the path analyses are depicted in **Table 3**. These correlations suggest significant positive associations of trait test anxiety and thought related test anxiety as well as exams’ personal value and thought related test anxiety at all three times of measure.

**Table 3:** Zero order correlations.

	(1)	(2)	(3)	(4)
**Pre-intervention**				
(1) Trait test anxiety				
(2) Exams’ personal value	0.164^∗^			
(3) Thought related test anxiety	0.553^∗∗^	0.262^∗∗^		
**Post-intervention**				
(4) Thought related test anxiety	0.612^∗∗^	0.262^∗∗^	0.676^∗∗^	
**Follow-up**				
(5) Thought related test anxiety	0.593^∗∗^	0.251^∗∗^	0.703^∗∗^	0.781^∗∗^

*N = 160. ^∗^*p* < 0.05, ^∗∗^*p* < 0.01*.

### Model 1: Combined Analysis

The fit statistics for model 1 were as follows, χ^2^(3) = 1.26, *p* = 0.739; CFI = 1.00; RMSEA = 0; SRMR = 0.03. Allover, a significant degree of the variance of thought related test anxiety measured at time 2 (*R*^2^ = 0.59, *SE* = 0.05, *p* < 0.001) and time 3 (*R*^2^ = 0.57, *SE* = 0.06, *p* < 0.001) was explained. All path coefficients are depicted in **Figure 1**. Trait test anxiety proved to be a significant positive predictor of thought related test anxiety at time 2 (β = 0.34, *SE* = 0.08, *p* < 0.001) and time 3 (β = 0.34, *SE* = 0.08, *p* < 0.001). The same was true for exams’ personal value regarding thought related test anxiety measured at time 2 (β = 0.09, *SE* = 0.05, *p* = 0.037), but not regarding thought related test anxiety measured at time 3 (β = 0.04, *SE* = 0.05, *p* = 0.215). As expected, we also found a significant effect of the dummy variable d1 (IBSR vs. control groups) on thought related test anxiety measured at time 2 (β = 0.19, *SE* = 0.05, *p* < 0.001) and at time 3 (β = 0.15, *SE* = 0.06, *p* < 0.001). The direction of the coefficients indicates that exploration of an individual worry thought with the IBSR technique is effective in reducing thought related test anxiety compared to reflecting on or distracting oneself from a worry thought.

### Model 2: Differential Analysis

The fit statistics for model 2 were as follows, χ^2^(6) = 1.58, *p* = 0.954; CFI = 1.00; RMSEA = 0; SRMR = 0.02. Model 2 explained a significant degree of the variance of thought related test anxiety measured at time 2 (*R*^2^ = 0.63, *SE* = 0.04, *p* < 0.001) and time 3 (*R*^2^ = 0.58, *SE* = 0.06, *p* < 0.001). All path coefficients are depicted in **Figure 2**. Again, trait test anxiety proved to be a significant positive predictor of thought related test anxiety at time 2 (β = 0.34, *SE* = 0.08, *p* < 0.001) and time 3 (β = 0.34, *SE* = 0.08, *p* < 0.001). The same was true for exams’ personal value regarding thought related test anxiety measured at time 2 (β = 0.08, *SE* = 0.05, *p* = 0.046), but not regarding thought related test anxiety measured at time 3 (β = 0.04, *SE* = 0.06, *p* = 0.245). Results also revealed a significant effect of the dummy variable d2a (IBSR vs. reflection) on thought related test anxiety measured at time 2 (β = 0.29, *SE* = 0.05, *p* < 0.001) and time 3 (β = 0.21, *SE* = 0.06, *p* < 0.001). The effect of the dummy variable d2b (IBSR vs. distraction) on thought related test anxiety measured at time 2 (β = 0.08, *SE* = 0.06, *p* = 0.084) and time 3 (β = 0.09, *SE* = 0.07, *p* = 0.088) was statistically non-significant. These results indicate that exploration of an individual worry thought with the IBSR technique is effective in reducing thought related test anxiety in comparison to reflecting on a worry thought. The β-values also indicated that IBSR was associated with lower thought related test anxiety than distraction, however, this effects was statistically non-significant.

### Effect Sizes

Means and standard deviations of thought related test anxiety are depicted separately for all three times of measure and all experimental conditions in **Table 4**. In a first step, we calculated standardized effect sizes (Cohen’s *d*) for the differences in thought related test anxiety between times 1 and 3 for each of the three experimental conditions, respectively. The results showed a high effect of the IBSR intervention (*d* = 0.96), a medium effect of distraction (*d* = 0.69), and a small effect of reflection (*d* = 0.40) according to Cohen’s (1988) standards. However, interpretation of these effect sizes is difficult due to different pre-intervention levels of thought related test anxiety and different group sizes. In a second step, we thus calculated the standardized effect size *d*_ppc2_, which is used for pre-test–post-test-control group designs (Morris, 2008). It takes full advantage of the available information and allows for a comparison between the experimental conditions. The results revealed a medium effect size when comparing the IBSR intervention with the reflection group at time 3 (*d*_pp2_ = 0.51) and a small effect size for the mean difference in thought related test anxiety between the IBSR intervention group and the distraction group at time 3 (*d*_pp2_ = 0.25).

**Table 4:** Means and standard deviations for thought related test anxiety.

	Pre-intervention		Post-intervention		Follow-up	
Groups	Mean	*SD*	Mean	*SD*	Mean	*SD*
IBSR Intervention	2.89	0.67	2.36	0.78	2.22	0.72
Distraction	2.79	0.71	2.41	0.84	2.29	0.74
Reflection	2.75	0.73	2.74	0.80	2.44	0.83

*IBSR intervention group: n = 53. Distraction group: n = 52. Reflection group: n = 55.*

## Discussion

In the present study, we investigated the effects of an IBSR short intervention on students’ test anxiety related to one individual worry thought choosing a twofold approach. In a first step, we tested if IBSR is more effective in reducing test anxiety than other strategies usually employed by test anxious students (combined analysis). We expected participants receiving the IBSR intervention to experience less test anxiety than participants of both control groups, who were either distracted from the worry thought or reflected on it. The results of the data analyses (model 1) confirmed this hypothesis. Participants who had explored their individual worry thought with IBSR reported significantly lower thought related test anxiety in comparison to participants of both control groups. This effect was found immediately after the experimental treatment and lasted at least till 2 days after the intervention. These results provide first evidence for the effectiveness of IBSR in reducing test anxiety regarding a specific and individual worry thought. We interpret the results of the present study according to the theoretical assumptions of control-value theory of achievement emotions (Pekrun, 2006), which states that test anxiety is caused by cognitive value and control appraisals. Therefore, test anxiety should decline due to an IBSR intervention that allows individuals to modify the cognitive appraisals by investigating their test anxiety causing worry thoughts. The results of our first analysis are also in line with previous research, which showed that participants of an IBSR intervention reported less anxiety than before the intervention (Leufke et al., 2013; Smernoff et al., 2015) or than an inactive control group (Krispenz and Dickhäuser, 2016). However, as mentioned, these previous studies lacked randomized assignment of participants to the IBSR group making causal interpretations of the results difficult. The results of our study significantly add to this literature in showing that the IBSR effects remain in a randomized control group research design.

In a second step, the two different control groups also allowed us to explore the effects of IBSR on thought related test anxiety in comparison to each of the control groups (distraction vs. reflection), respectively, (differential analysis). In particular, we wanted to investigate the effects, the IBSR intervention would have in comparison to *distraction* from the worry thought. Because distraction primarily works through the use of attentional deployment (Thiruchselvam et al., 2011), but is not assumed to alter the interpretation of the worry thought (Rost and Schermer, 2007), we expected distraction to have at least a short-term effect on thought related test anxiety, but we had no prediction how pronounced the anxiety reducing effect of distraction would be in comparison to IBSR. Results numerically showed that IBSR had a small effect (*d*_pp2_ = 0.25) in comparison to distraction from the worry thought, even though this effect was statistically non-significant. This was not only true for the post measurement, but also regarding the 2-day follow up. These results are partly in line with research, which shows that distraction is effective in reducing emotional responding *short term* (Thiruchselvam et al., 2011) when individuals are confronted with unpleasant emotional stimuli (e.g., negative images). However, according to Thiruchselvam et al. (2011) distraction usually leads to a more emotional arousal upon re-exposure to the same stimuli. Yet, in the present study the anxiety reducing effect of distraction was still present 2 days after the experimental treatment. This could be due to methodological differences – while in the study of Thiruchselvam et al. (2011) participants upon re-exposure were instructed to *not* distract themselves, in our study participants of the distraction group were not given any similar instruction. Thus, participants of the distraction control group might have remembered and applied the distraction strategy they had learned in the experimental setting 2 days after the experiment. However, results of the differential analysis also showed that IBSR was statistically significantly more effective than *reflection* in reducing thought related test anxiety with a medium effect size (*d*_pp2_ = 0.51).

Also, results revealed that 2 days after the experimental intervention levels of thought related test anxiety had decreased for all three groups indicating a systematic effect of time regardless of experimental condition. We assume that this effect is due to the fact that the follow-up measures were assessed not in the experimental laboratory but online. Even though participants interacted in both events with a computer (rather than an experimenter), the online anxiety assessment might have been different relative to the assessment in the experimental lab. In the laboratory session, participants had to reflect for about 15 min on the upcoming exams (including thinking of the upcoming academic exams, describing their most frightening exam in detail, rating the exams’ personal value and the respective anxiety-level). Also, they deeply elaborated what worried them most about their most frightening exam. In contrast, at the follow-up measure participants were only instructed to remember their most frightening exam and their individual worry thought before thought related test anxiety was assessed. Thus, we assume that in the online measure the upcoming exam was not as salient to participants as in the experimental setting. Thus, the drop of thought related test anxiety for all groups might be a consequence of the setting of assessment. However, it is also possible that the distance of time had discharged state anxiety. Nevertheless, the drop in thought related test anxiety seems to be universal and therefore does not limit the interpretation of the results.

### Limitations

An important limitation of the present study is the short-term measurement of the found effect. Data was collected before, immediately, and again 2 days after the experimental treatment. Thus, the data provides preliminary evidence for the effectiveness of IBSR in reducing test anxiety regarding a specific and individual worry thought. Accordingly, future studies should involve a longer follow-up period to investigate if the found effects also hold long-term.

Further, the IBSR technique is usually taught in a group setting and with the help of a coach when participants are new to the method. In our study, the procedure was different as participants practiced the IBSR method on the computer without any further direct assistance of the experimenter. When being asked about their experience using the IBSR method, none of the IBSR participants reported problems when answering the four questions (and respective sub-questions). However, some participants reported difficulties finding examples when applying the IBSR turnaround technique. These difficulties might be a result of participants’ confirmation bias (Nickerson, 1998) because the turnaround technique challenges participants’ initial belief. This might be a hint that participants new to IBSR would profit from assistance when applying the IBSR turnaround technique for the first time.

### Practical Implications

Our findings have practical implications for educational settings. Test anxiety can lead to undesirable consequences for learning, motivation, and academic achievement (e.g., Cassady and Johnson, 2002). Our results provide first evidence that IBSR is helpful in reducing state test anxiety in a non-clinical sample of university students. The found effects were small (IBSR vs. distraction: *d*_pp2_ = 0.25) to moderate (IBSR vs. reflection: *d*_pp2_ = 0.51). We consider the effects to be clinically significant and practically relevant for the following reasons. Firstly, the IBSR intervention tested in the present study lasted only 20 min. According to the meta-analysis of Ergene (2003), similar test anxiety *short* interventions (i.e., with therapy times of 0–60 min) have small effects (*E*_+_ = 0.34). Secondly, regarding *individual* (i.e., non-group) interventions, Ergene (2003) also found just a small mean effect of *E*_+_ = 0.34. Thirdly, interventions using *cognitive approaches* to reduce test anxiety have a moderate effect (*E*_+_ = 0.63). Note, that the studies reported by Ergene (2003) obtained effects over a much longer period of time than the present study did.

In light of these results, the IBSR short intervention can be considered a practical tool to reduce test anxiety in students for the following reasons: Firstly, participants of the present study merely investigated one worry thought. Effects of the IBSR intervention might be stronger the more worry thoughts associated with a specific testing situation are explored with the IBSR method. Secondly, due to its standardized procedure the IBSR intervention allows for a structured way of self-inquiry without the need for a therapist. This makes the IBSR method an individually applicable technique for test anxious students without the need for a high investment of time and effort. Teachers and other professionals working in the educational context might thus consider implementing elements of the IBSR method when preparing test anxious students for their exams to help them in dealing with their worry thoughts in a structured and functional way.

## Conclusion

Our findings provide first evidence for the effectiveness of an IBSR short intervention in reducing test anxiety in university students in an experimental setting. Our results suggest that university students can apply IBSR to identify and self-investigate their individual worry thoughts associated with a specific frightening exam to reduce their test anxiety as an alternative strategy to distraction from or mere reflection of such worry thoughts.

## Ethics Statement

By the time we conducted the study and acquired the data, it was neither compulsory nor customary at the University of Mannheim to seek explicit ethical approval for an experimental study including only participants’ self-reports on test anxiety. However, we carefully ensured that the study was conducted in line with the ethical guidelines of the American Psychological Association (APA) and in full accordance with the Ethical Guidelines of the German Association of Psychologists (DGPs): (1) We did not induce test anxiety or any other negative states in the participants but merely assessed their thoughts and affect regarding their upcoming exams. We thus had no reasons to assume that our study would induce any negative states in the participants exceeding the normal risks of studying at a university and preparing for exams. (2) AK is now working at the University of Bern, Switzerland. At this university, she is conducting a follow-up study, which explicitly targets participants with test anxiety and/or procrastination (in the present study, participants were acquired from a non-clinical sample). In the new study, participants receive nine hours of IBSR training (in the present study, the IBSR intervention lasted only 20 min). The human research ethics committee of the University of Bern just recently approved this new study. This can be considered as a clear retrospective sign that there are no ethical concerns with regard to the procedure of the present study. (3) The study exclusively made use of anonymous questionnaires. The separate questionnaires were matched for the analyses by relying on codenames. No identifying information was obtained from participants. Written informed consent was obtained according to the guidelines of the German Research Association (DFG). Informed consent included information about (1) research object, (2) study procedure, (3) duration and allowance, (4) possible benefits of participation, (5) anonymity of data collection, and (6) possible risks of participation. Also, participants were explicitly informed that participation was voluntary and could be terminated at any time without any reason or negative consequences for the participant. Participants had to declare that they were at least 18 years old, had read the informed consent information, and agreed to the rules of participation.

## Author Contributions

AK and OD contributed meaningfully to the paper. AK developed the study concept and design. AK and OD analyzed and interpreted the data. AK prepared the draft manuscript and OD provided critical revisions. AK and OD approved the final version to be published and agreed to be accountable for all aspects of the work in ensuring that questions related to the accuracy or integrity of any part of the work are appropriately investigated and resolved.

## Conflict of Interest Statement

The authors declare that the research was conducted in the absence of any commercial or financial relationships that could be construed as a potential conflict of interest.

## Footnotes

1 Please note that the mean values indicate that the majority of participants reported to feel anxious about the upcoming exam. However – and in line with the fact that the sample was not recruited as a sample of test-anxious individuals – an inspection of the empirical range showed that a least some of the participants reported low levels of anxiety. Instead of deleting these participants from the sample we decided to run the analyses with the full sample in order to allow for a more conservative and externally more valid estimation of the effects of IBSR in non-clinical samples.
