# Corrigendum: Effects of an Inquiry-Based Short Intervention on State Test Anxiety in Comparison to Alternative Coping Strategies

**DOI:** 10.3389/fpsyg.2018.02734

**Published:** 2019-01-10

**Authors:** Ann Krispenz, Oliver Dickhäuser

**Affiliations:** School of Social Sciences, Department of Psychology, University of Mannheim, Mannheim, Germany

**Keywords:** educational psychology, test anxiety, cognitive appraisals, inquiry-based stress reduction, short intervention

In the original article, there was a mistake in Figure 1 and Figure 2 as published. The coefficients for the effects of the experimental intervention condition on thought related test anxiety were mistakenly reported as negative even though the coefficients for these effects are positive. The corrected Figure 1 and Figure 2 appear below.

**Figure 1:**
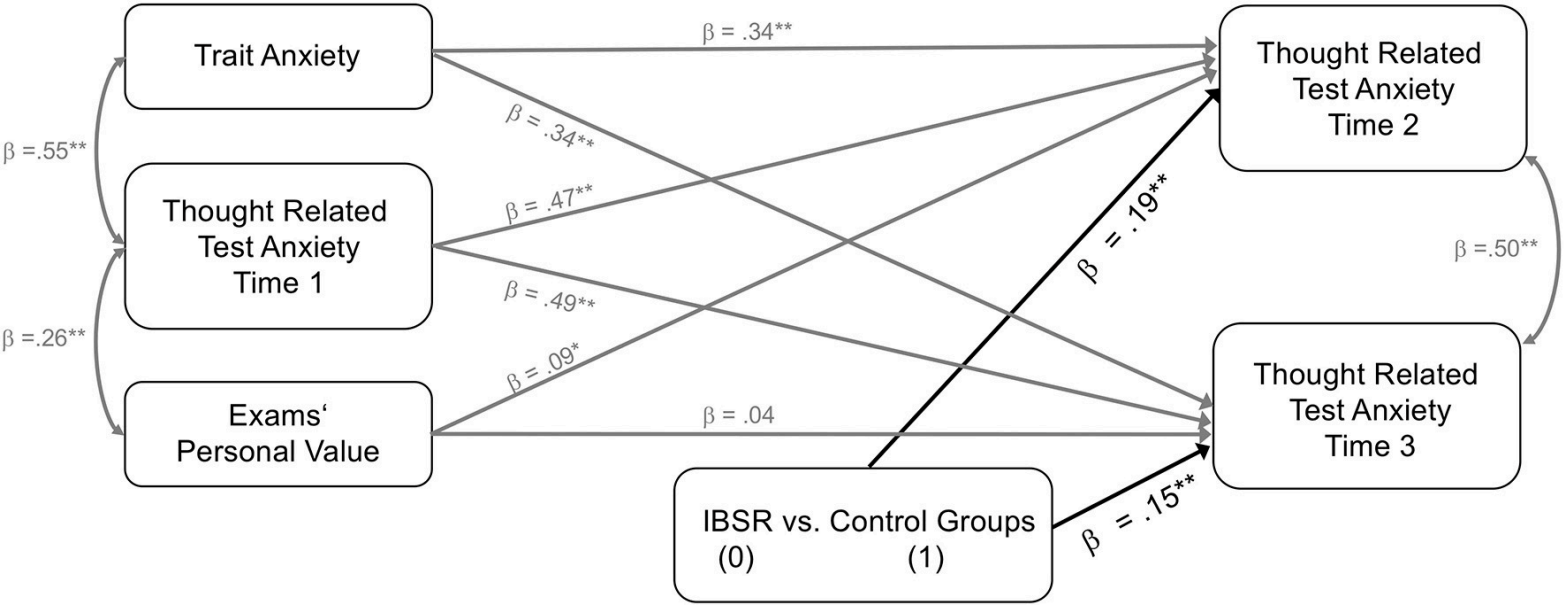
Experimental intervention effects on targeted thought related test anxiety at times 2 and 3 while controlling for initial scores of thought related test anxiety, trait test anxiety, and exams' personal value. IBSR (dummy coded 0) vs. distraction and reflection group (both dummy coded 1). All parameter estimates are standardized. *N* = 160. ^*^p ≤ 0.05, ^**^p ≤ 0.01.

**Figure 2:**
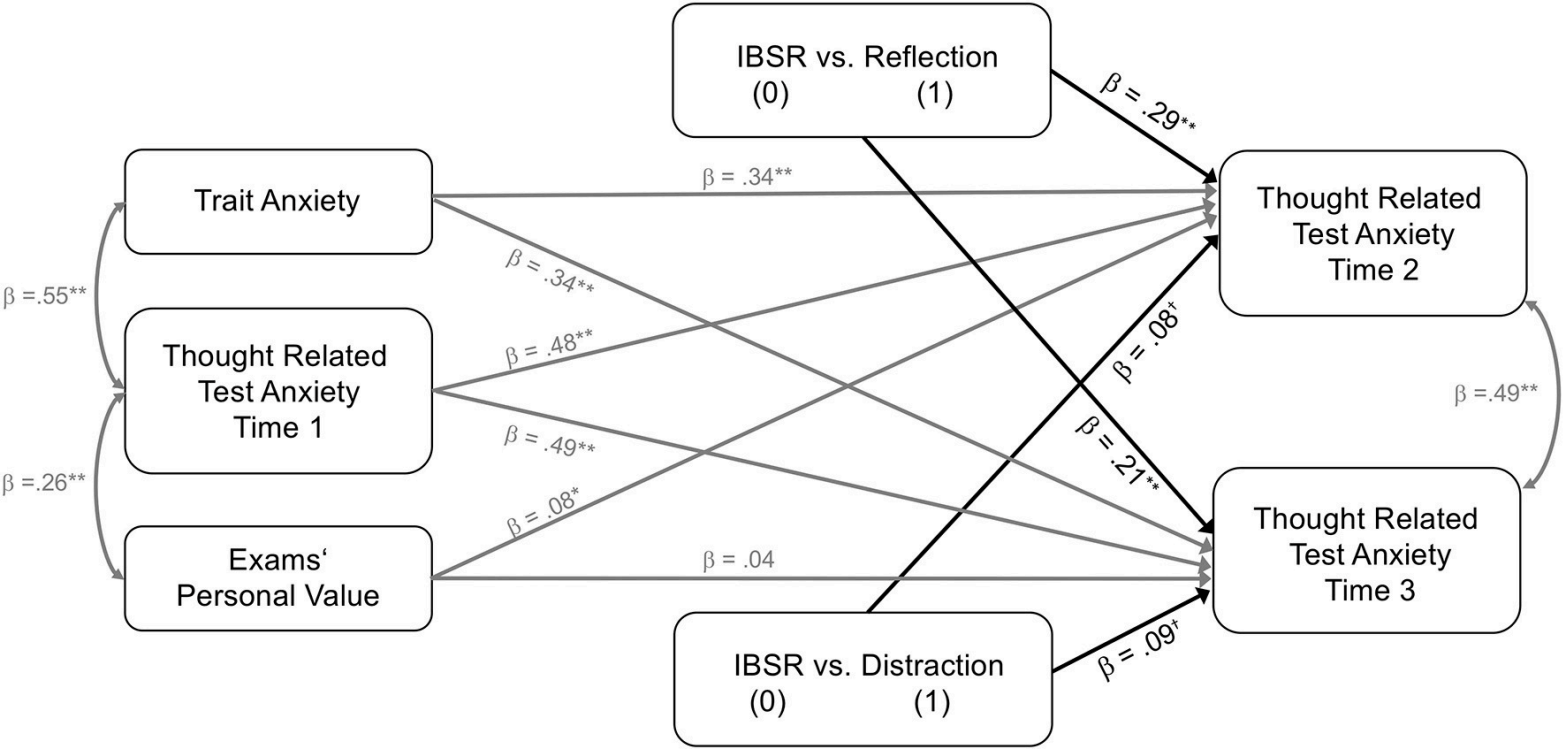
Experimental intervention effects on targeted thought related test anxiety at times 2 and 3 while controlling for pre-intervention scores of thought related test anxiety, trait test anxiety, and exams' personal value. IBSR vs. the reflection (IBSR dummy coded = 0, reflection dummy coded = 1, distraction dummy coded 0); IBSR vs. the distraction (IBSR dummy coded = 0, reflection dummy coded = 0, distraction dummy coded 1). All parameter estimates are standardized. *N* = 160. †*p* ≤ 0.10, ^*^p ≤ 0.05, ^**^p ≤ 0.01.

Because of the error mentioned above, a correction has been made to the **Results, Model 1: Combined Analyses**:

“The fit statistics for model 1 were as follows, χ^2^(3) = 1.26, *p* = 0.739; CFI = 1.00; RMSEA = 0; SRMR = 0.03. Allover, a significant degree of the variance of thought related test anxiety measured at time 2 (*R*^2^ = 0.59, *SE* = 0.05, *p* < 0.001) and time 3 (*R*^2^ = 0.57, *SE* = 0.06, *p* < 0.001) was explained. All path coefficients are depicted in Figure 1. Trait test anxiety proved to be a significant positive predictor of thought related test anxiety at time 2 (β = 0.34, *SE* = 0.08, *p* < 0.001) and time 3 (β = 0.34, *SE* = 0.08, *p* < 0.001). The same was true for exams' personal value regarding thought related test anxiety measured at time 2 (β = 0.09, *SE* = 0.05, *p* = 0.037), but not regarding thought related test anxiety measured at time 3 (β = 0.04, *SE* = 0.05, *p* = 0.215). As expected, we also found a significant effect of the dummy variable d1 (IBSR vs. control groups) on thought related test anxiety measured at time 2 (β = 0.19, *SE* = 0.05, *p* < 0.001) and at time 3 (β = 0.15, *SE* = 0.06, *p* < 0.001). The direction of the coefficients indicates that exploration of an individual worry thought with the IBSR technique is effective in reducing thought related test anxiety compared to reflecting on or distracting oneself from a worry thought.”

A correction has also been made to the **Results, Model 2: Differential Analyses**:

“The fit statistics for model 2 were as follows, χ^2^(6) = 1.58, *p* = 0.954; CFI = 1.00; RMSEA = 0; SRMR = 0.02. Model 2 explained a significant degree of the variance of thought related test anxiety measured at time 2 (*R*^2^ = 0.63, *SE* = 0.04, *p* < 0.001) and time 3 (*R*^2^ = 0.58, *SE* = 0.06, *p* < 0.001). All path coefficients are depicted in Figure 2. Again, trait test anxiety proved to be a significant positive predictor of thought related test anxiety at time 2 (β = 0.34, *SE* = 0.08, *p* < 0.001) and time 3 (β = 0.34, *SE* = 0.08, *p* < 0.001). The same was true for exams' personal value regarding thought related test anxiety measured at time 2 (β = 0.08, *SE* = 0.05, *p* = 0.046), but not regarding thought related test anxiety measured at time 3 (β = 0.04, *SE* = 0.06, *p* = 0.245). Results also revealed a significant effect of the dummy variable d2a (IBSR vs. reflection) on thought related test anxiety measured at time 2 (β = 0.29, *SE* = 0.05, *p* < 0.001) and time 3 (β = 0.21, *SE* = 0.06, *p* < 0.001). The effect of the dummy variable d2b (IBSR vs. distraction) on thought related test anxiety measured at time 2 (β = 0.08, *SE* = 0.06, *p* = 0.084) and time 3 (β = 0.09, *SE* = 0.07, *p* = 0.088) was statistically non-significant. These results indicate that exploration of an individual worry thought with the IBSR technique is effective in reducing thought related test anxiety in comparison to reflecting on a worry thought. The β-values also indicated that IBSR was associated with lower thought related test anxiety than distraction, however, this effects was statistically non-significant.”

The authors apologize for these errors and state that this does not change the scientific conclusions of the article in any way. The original article has been updated.

## Conflict of Interest Statement

The authors declare that the research was conducted in the absence of any commercial or financial relationships that could be construed as a potential conflict of interest.

